# Supplementary material for: Muscle‐derived exophers promote reproductive fitness
Source: EMBO Rep. 2021 Jul 20;22(8):e52071. doi: 10.15252/embr.202052071 (PMC8339713; doi:10.15252/embr.202052071)
Supplement: Supplementary file 1 — Appendix [file EMBR-22-e52071-s003.pdf]

Appendix

Appendix Table S1. Percentage of animals with exophers. 2
Appendix Table S2. List of all C. elegans strains used in this study. 4
Appendix Table S3. List of all oligonucleotides strains used in this study. 5

Appendix Table S1. Percentage of animals with exophers.

Figure number	Condition	Percentage of animals with exophers
Figure 1F	Control	100%
Figure 1H	Control	97%
	atg-7 RNAi	89%
	lgg-1 RNAi	95%
Figure 1I	Control	93%
	hsf-1 RNAi	93%
Figure 1J	Oxidative stress +	97%
	Oxidative stress -	100%
	Heat stress +	97%
	Heat stress -	90%
Figure 2A	Hermaphrodite day 0	0%
	Hermaphrodite day 1	69%
	Hermaphrodite day 2	98%
	Hermaphrodite day 3	92%
	Hermaphrodite day 4	84%
	Hermaphrodite day 5	79%
	Hermaphrodite day 6	78%
	Hermaphrodite day 7	72%
	Hermaphrodite day 8	73%
	Hermaphrodite day 9	74%
	Hermaphrodite day 10	84%
	Hermaphrodite day 11	78%
	Hermaphrodite day 12	82%
	Hermaphrodite day 13	93%
	Hermaphrodite day 14	86%
	Hermaphrodite day 15	88%
	Male day 0	0%
	Male day 1	1%
	Male day 2	5%
	Male day 3	7%
	Male day 4	33%
	Male day 5	31%
	Male day 6	32%
	Male day 7	35%
	Male day 8	36%
	Male day 9	58%
	Male day 10	64%
	Male day 11	55%
	Male day 12	62%
	Male day 13	54%
	Male day 14	58%
	Male day 15	70%

Figure 2B	15°C without offspring	40%
	15°C with offspring	79%
	25°C without offspring	42%
	25°C with offspring	100%
Figure 2D	Control	95%
	FUdR	15%
Figure 3A	Control	89%
Figure 3B	Control	93%
	goa-1 RNAi	87%
	egl-1 RNAi	98%
	egl-4 RNAi	98%
Figure 3C	Control L4 → AD1	26%
	Starved L4 → AD1	16%
	Control AD2 → AD3	80%
	Starved AD2 → AD3	98%
Figure 3E	Control	94%
	Embryo-conditioned buffer	95%
Figure 3F	Control	96%
	emb-27 RNAi	93%
Figure 4E	Control	90%
	vit-2 RNAi	100%
Figure 4G	Control	98%
	rme-2 RNAi	34%
Figure S1B	Control	96%
	emb-8 RNAi	93%
	pod-1 RNAi	89%
Figure S2A	Control (H)	95%
	Embryos lysate (H)	100%
	Larvae lysate (H)	100%
	Control (M)	9%
	Embryos lysate (M)	6%
Figure S2B	Control	97%
	egl-1 RNAi	98%
	Control + FUdR	33%
	egl-1 RNAi + FUdR	29%
Figure S2D	Control	100%
	unc-45 RNAi	71%
	Control	93%
	unc-54 RNAi	98%
Figure S4A	Control	96%
	ced-1 RNAi	99%
	ced-6 RNAi	96%

Appendix Table S2. List of all *C. elegans* strains used in this study.

Strain	Genotype	Usage	Reference
N2	Wild type	Figure 3E	Brenner 1974
ACH91	*wacIs6[myo-3p::pas-7::GGGGS Linker-wrmScarlet::unc-54 3'UTR, unc-119(+)], wacIs14[myo-3p::tomm-20_1-50aa::attB5::mGFP::unc-54-3'UTR, unc-119(+)]*	Figure 1A, J; Figure 4E Figure S2D	Generated for this study
ACH93	*wacIs1[myo-3 promoter::rpn-5 CAI=0.97::GGGGS Linker-wrmScarlet::unc-54 3'UTR, unc-119(+)], wacIs14[myo-3 promoter::tomm-20_1-50aa::attB5::mGFP::unc-54-3'UTR, unc-119(+)]*	Figure 1A-B, E-I; Figure 2A, D-E; Figure 3A-C, E-F; Figure 4B-D, G, I; Figure S1A-B; Figure S2A-C Figure S3 Figure S4A	Generated for this study
ACH199	*wacIs1[myo-3 promoter::rpn-5 CAI=0.97::GGGGS Linker-wrmScarlet::unc-54 3'UTR, unc-119(+)], vit-2(crg9070[vit-2::gfp]) X*	Figure 1C, Figure 4F,H; Figure S1C-D Figure S4B-C	Generated for this study
AGD885	*rrf-3(b26) II; fem-1(hc17) IV; uthEx633 [myo-3p::GFP]*	Figure 2B-C	Vilchez et al. 2012
TUR5	*wacIs1[myo-3 promoter::rpn-5 CAI=0.97::GGGGS Linker-wrmScarlet::unc-54 3'UTR, unc-119(+)], wacIs14[myo-3 promoter::tomm-20_1-50aa::attB5::mGFP::unc-54-3'UTR, unc-119(+)], wwaEx2[unc-122 promoter::GFP]*	Figure 1D	Generated for this study
CB4088	*him-5(e1490)V*	Figure 2B	Caenorhabditis Genetics Centre

Appendix Table S3. List of all oligonucleotides strains used in this study.

OLIGONUCLEOTIDE	SOURCE	IDENTIFIER
MT38_pCG150trimmed_Fwd_3UTRunc-54<br>5’-AACTGTTTATAATTCACTGGCCGTCGTTTTAC-3’	Sigma-Aldrich	N/A
MT47_3UTRunc-54_Rev_pCG150trimmed<br>5’-TGAATTATAAACAGTTATGTTTGGTATATTGG-3’	Sigma-Aldrich	N/A
MT39_pCG150trimmed_Rev_myo-3prom<br>5’-TAATCACTATAGCTTGGCGTAATCATGGTCAT-3’	Sigma-Aldrich	N/A
MT40_myo-3prom_Fwd_pCG150trimmed<br>5’-CAAGCTATAGTGATTATAGTCTCTGTTTTCGT-3’	Sigma-Aldrich	N/A
MT62_tomm-20_Fwd_myo-3prom<br>5’-atccatctagaaGTTaaaaATGTCAGATACCATTCTCGGA-3’	Sigma-Aldrich	N/A
MT63_tomm-20_Rev_GFP<br>5’-CCCTTGGATTCAACTTTTGTATACAAAGTTGTGGC-3’	Sigma-Aldrich	N/A
MT64_GFP_fwd_tomm-20<br>5’-AAGTTGAATCCAAGGGAGAGGAGCTC-3’	Sigma-Aldrich	N/A
MT65_GFP_Rev_unc-54-3UTR<br>5’-gagtaattggacGTTTTACTTGTAGAGCTCGTCCATTC-3’	Sigma-Aldrich	N/A
MT66 rpn-5 Fwd myo-3<br>5’-atccatctagaaGTTaaaaATGGCAGACAGACGC-3’	Sigma-Aldrich	N/A
MT76_rpn-5_rev_OL-wrmScarlet<br>5’-GGATCCACCACCTCCGGCACGTGGGGCG-3’	Sigma-Aldrich	N/A
MT74_OL-wrmScarlet_fwd 5’-<br>GGAGGTGGTGGATCCGGAGGTGGTGGATCCGGAGGAGGAGGATC<br>Cgtcagcaagggagaggc-3’	Sigma-Aldrich	N/A
MT69 wrmScarlet Rev unc-54<br>5’-gagtaattggacGTTTTACTTGTAGAGCTCGTCCATTC-3’	Sigma-Aldrich	N/A
MT72_pas-7_fwd_myo-3_prom<br>5’-atccatctagaaGTTatgagttcaatcggtaccgg-3’	Sigma-Aldrich	N/A
MT73_pas-7_rev_OL-wrmScarlet<br>5’-GGATCCACCACCTCCttcaacttttgtatacaaagttgtatcgtc-3’	Sigma-Aldrich	N/A
