# Supplementary material for: Muscle‐derived exophers promote reproductive fitness
Source: EMBO Rep. 2021 Jul 20;22(8):e52071. doi: 10.15252/embr.202052071 (PMC8339713; doi:10.15252/embr.202052071)
Supplement: Supplementary file 2 — Expanded View Figures PDF [file EMBR-22-e52071-s006.pdf]

Expanded View Figures

Figure EV1. Regulation and ultrastructure of muscular exophers.

A Small fraction of muscular exophers contain mitochondria. $n = 60$; $N = 6$. n represents the number of worms; N represents the number of experimental repeats that were combined into a single value.

B The formation of muscle-derived exophers, like that of neuronal exophers, is affected by the depletion of EMB-8 and POD-1. $n = 80–120$; *pod-1* RNAi – $N = 2$, *emb-8* RNAi – $N = 3$. Data are shown as mean ± SEM; n represents the number of worms; N represents the number of experimental repeats combined into a single value; ****$P < 0.0001$, Mann–Whitney test.

C, D Exophers visualization using correlative light electron microscopy (CLEM). Exopher's red fluorescence (red arrow). (C′, D′) Exopher (red arrow) surrounded by marks (asterisk) after NIRB, autofluorescence of branded frame edges is visible. (C″, D″) CLEM tracking the exopher—LM image, EM image, and their overlay. (C‴, D‴) Image shows exopher's (colored in red) ultrastructure.

Data information: Thick, gray arrows show an order of steps done for the final exopher detection under the electron microscope. Red arrows point to exophers; asterisks and colored points show landmarks after NIRB (Near Infra-Red Branding), which are used for correlating fluorescence and electron microscopy pictures. Scale bars are: C–C′, D–D′, C″ (bottom picture), D″ (bottom picture)—10 μm; C″ (top and middle picture), D″ (top and middle picture)—50 μm; C‴, D‴—1 μm.

 © 2021 The Authors

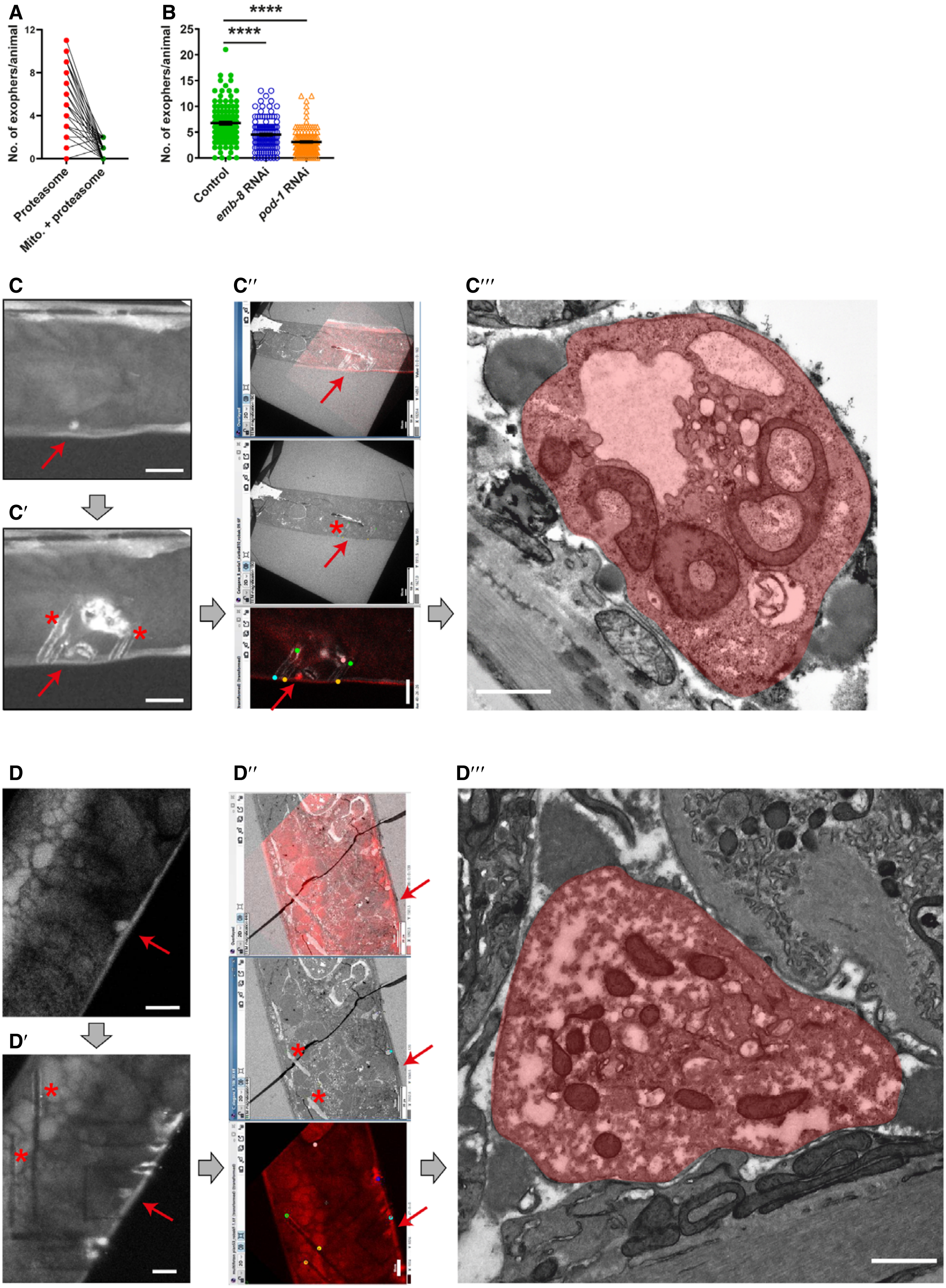
A
B
C
C′
C″
C‴
D
D′
D″
D‴
No. of exophers/animal
Proteasome
Mito. + proteasome
Control
emb-8 RNAi
pod-1 RNAi
****
****

Figure EV1.

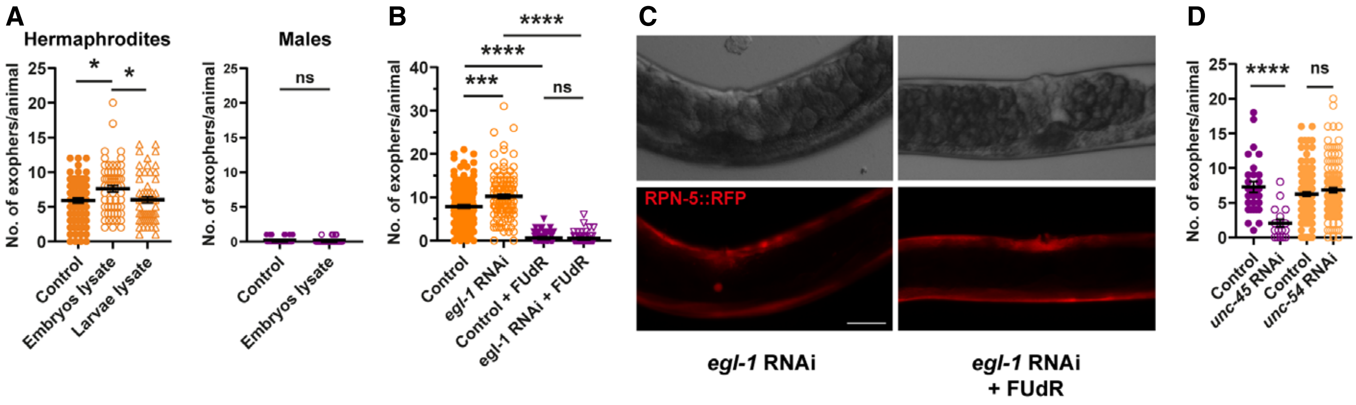

Figure EV2. Signal derived from *in utero*-developing *embryos activates exopheresis in hermaphrodites.*

A Exposure of young adult hermaphrodites but not males to lysed embryos increases exopher production. Hermaphrodites: n = 57–74; N = 3. Males: n = 90 and 80; N = 2.

B Retention of dead embryos in the hermaphrodite uterus does not increase exopher production. n = 112–178; N = 6 and 3.

C Representative images of the mid-body in panel B. Scale bar is 50 µm.

D Disturbance of myosin thick filament assembly in the whole animal (*unc-45* RNAi) but not specifically in BWM (*unc-54* RNAi) leads to decreased exopher production. n = 17–121; N = 2 and 3.

Data information: Data are shown as mean ± SEM; n represents number of worms; N represents number of experimental repeats that were combined into a single value; ns—not significant, * $P < 0.05$, *** $P < 0.001$, **** $P < 0.0001$; (A, B, D) Mann–Whitney test.

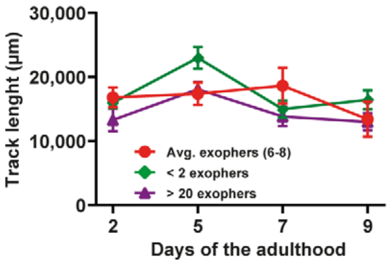

Figure EV3. Active exopheresis does not affect worm mobility.

The graph shows the track lengths of animals exhibiting different exopheresis activities. n = 11–38; N = 3. Data are shown as mean ± SEM.

Source data are available online for this figure.

 © 2021 The Authors

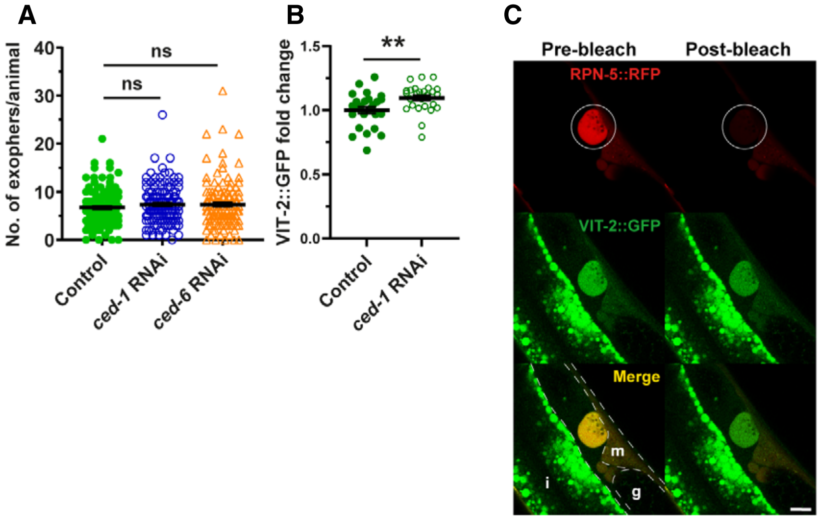

Figure EV4. Exophers contain muscle-produced vitellogenin.

A Muscular exophers formation does not depend on the apoptotic engulfment pathway proteins CED-1 and CED-6. n = 114–120; N = 3.

B CED-1 depletion leads to a small increase in VIT-2::GFP in embryos. n = 25 and 26; N = 2.

C Images show the formation of an exopher filled with the proteasome and vitellogenin. Images were captured before and after RPN-5::RFP photobleaching, confirming that the high signal from endogenous VIT-2::GFP in forming exophers is not an imaging artifact. The white circle marks the bleached area using a 555-nm laser and the position of the developing exopher. Dashed lines mark different tissue borders: m—muscle, i—intestine, g—gonad. Scale bar is 10 μm.

Data information: Data are shown as mean ± SEM; n represents the number of worms; N represents the number of experimental repeats that were combined into a single value; ns—not significant ** $P < 0.01$; (A, B) Mann–Whitney test.

© 2021 The Authors
